# Effects of equine SALSA on neutrophil phagocytosis and macrophage cytokine production

**DOI:** 10.1371/journal.pone.0264911

**Published:** 2022-03-14

**Authors:** Gary Kwok Cheong Lee, Heng Kang, Janet Beeler-Marfisi, William Sears, Brandon N. Lillie, Dorothee Bienzle

**Affiliations:** 1 Department of Pathobiology, University of Guelph, Guelph, Ontario, Canada; 2 Department of Population Medicine, University of Guelph, Guelph, Ontario, Canada; Michigan State University, UNITED STATES

## Abstract

Salivary scavenger and agglutinin (SALSA) is a secreted protein with various immunomodulatory roles. In humans, the protein agglutinates and inactivates microorganisms, and inhibits the release of pro-inflammatory cytokines. Saliva, which is rich in SALSA, accelerates bacterial phagocytosis, but SALSA’s contribution is unclear. In horses, the functions of SALSA in inflammation remain undetermined, so they were investigated through phagocytosis and cytokine assays. Equine SALSA was purified from duodenal tissue, which contains abundant SALSA. To assess phagocytosis, fluorescently-labelled bacteria were incubated with 20, 10, 5, or 2.5 μg/mL of SALSA or phosphate buffered saline (PBS), and then incubated at 37°C or on ice with whole blood from seven healthy horses. Fluorescence was measured by gating on neutrophils using a flow cytometer, and compared between groups. To assess effects on cytokine production, alveolar macrophages were isolated from bronchoalveolar lavage fluid of five healthy horses and cultured in serum-free media for 24 hours with different concentrations of SALSA plus 1 μg/mL lipopolysaccharide (LPS), only LPS, or only media. Cytokines were measured in supernatant using an equine-specific multiplex bead immunoassay. There was significantly greater phagocytosis in samples incubated at 37°C compared to incubation on ice. Samples incubated with 20 μg/mL of SALSA at 37°C had less phagocytosis compared to samples with 10 or 2.5 μg/mL SALSA, or PBS. Alveolar macrophages incubated with SALSA plus LPS released significantly less CXC motif chemokine ligand 1, interleukin-8, interleukin-10, and tumor necrosis factor α, and more granulocyte colony stimulating factor (G-CSF), compared to macrophages incubated with LPS alone. These findings indicate anti-inflammatory effects, which may be due to interference with toll-like receptor 4 recognition of LPS or downstream signaling. Increase in G-CSF following incubation with SALSA suggests a novel mechanism for immunoregulation of alveolar macrophages by SALSA, addressing a knowledge gap regarding its functions in horses.

## Introduction

Salivary scavenger and agglutinin (SALSA), also known as “deleted in malignant brain tumors 1” (DMBT1), gp340, and salivary agglutinin (SAG), is a multifunctional protein that is mainly secreted by mucosal epithelial cells [1, 2]. The protein is considered to primarily modulate innate immunity, inflammation, and epithelial homeostasis. Frequent loss or reduction in gene expression has been noted in various neoplasms, suggesting also a role in tumor suppression, but this has been an inconsistent finding, and direct evidence linking gene deletion and tumorigenesis is lacking [3]. Mucosal surfaces are the interface of encounters between most pathogens and hosts, and at these sites SALSA binds and inactivates microorganisms, and interacts with ligands on leukocytes and epithelial cells to regulate inflammation [1]. Such ligands include secretory IgA, surfactant proteins A and D, lactoferrin, trefoil factors and complement components [1, 4–7].

The SALSA protein is composed of scavenger receptor cysteine-rich (SRCR) domains, “C1r/C1s, urchin embryonic growth factor and bone morphogenetic protein-1” (CUB) domains, and a zona pellucida (ZP) domain. The highly homologous SRCR domains are a feature of a variety of secreted molecules known as the SRCR protein superfamily, and are key to SALSA’s ability to bind a variety of ligands, including bacterial motifs through a dual cation-binding site [2]. Binding to bacteria leads to their agglutination, inactivation, and removal [8]. Saliva, which is rich in SALSA, enhances aggregation of bacteria, and subsequent acceleration of phagocytosis by granulocytes [9, 10]. However, the contribution of SALSA to phagocytosis remains unclear. Recognition of bacterial motifs by inflammatory cells induces a cascade of events including phagocytosis, microbial killing and production of cytokines. For example, binding of lipopolysaccharide (LPS) to the Toll-like receptor 4 (TLR4)- myeloid differentiation factor (MD)-2 complex leads to the activation of signaling components such as nuclear factor kappa-light-chain-enhancer of activated B cells (NF-κB), and the production of pro-inflammatory cytokines [11]. Recombinant human SALSA inhibited LPS-induced activation of the NF-κB pathway and release of interleukin 8 (IL-8) by both epithelial and myeloid cells [8]. The molecular determinants underpinning this interaction remain to be clearly defined.

In horses, as in humans, SALSA primarily localized to epithelial cells of mucosal surfaces, and intracellularly was concentrated at the cell apex, typical of secreted molecules [12]. The structure of equine SALSA is similar to that in other species and consists of SRCR, CUB, and ZP domains [12]. There is individual variation in the number of SRCR domains, which is consistent with different isoforms, a fact that has also been noted in humans [12]. Decreased SALSA gene expression was noted in bronchial biopsies of horses affected by severe equine asthma [12–14]. Severe equine asthma (SEA) is a debilitating condition of mature horses associated with systemic inflammation [15]. Downregulation of the SALSA gene in SEA has been suggested to reflect immune dysregulation, which in turn may contribute to perpetuating inflammation. However, so far knowledge about the actual function of equine SALSA is limited [12, 16]. Evaluating the effects of SALSA on cytokine release by alveolar macrophages would expand our understanding of the role of SALSA in the equine respiratory tract. While SEA is not an infectious disease, it is initiated and exacerbated by a variety of organic and inorganic microscopic matter, including components of bacteria and fungi [17]. Therefore, phagocytosis may play an important role in the initial phases of the disease, and serves as the precursor to immune responses including cytokine production. Investigation of the effects of SALSA on phagocytosis would improve our understanding of its immunomodulatory functions in horses. The effects on phagocytosis, to the best of our knowledge, have not been studied in any species.

The objectives of this study were to determine the effects of SALSA on equine leukocytes. It was hypothesized that equine SALSA would increase bacterial phagocytosis by neutrophils and inhibit inflammatory cytokine production by alveolar macrophages. The overarching aims were to address a knowledge gap regarding the immunomodulatory functions of SALSA in horses.

## Materials and methods

### Purification of SALSA by immunoprecipitation

A tissue lysate was prepared from fresh equine duodenum collected at the autopsy of a 12-year-old Standardbred gelding euthanized due to orthopedic disease. The horse was submitted for a routine postmortem examination, and was not euthanized specifically for this study. Duodenum was selected for protein purification since SALSA is highly expressed in this tissue [1, 12]. For tissue lysis, 50 mg of tissue were combined with 1 mL of lysis buffer (CelLytic^™^ MT Cell Lysis Reagent; Sigma-Aldrich, St Louis, MO, USA) and 10 μL of a protease inhibitor cocktail (P8340; Sigma-Aldrich), and disrupted in a TissueLyser II apparatus (Qiagen, Hilden, Germany). The lysed contents were centrifuged for 10 minutes at 4°C and 16 000 x g. The supernatant was collected as it contained soluble proteins. Supernatant (150 μg of protein extract, determined by spectrophotometry; Nanodrop 2000, Thermo Fisher Scientific) was incubated with 1 μL of a 1:100 dilution of DMBT1 antibody (polyclonal rabbit IgG, concentration 1 μg/μL, RRID: AB_2810221, Sino Biological, Wayne, PA, USA) on ice for 2 hours. This antibody was previously validated for horses [12]. Then, 100 μL of protein A-coated magnetic microbeads (μMACS protein A microbeads; Miltenyi Biotec, Auburn, CA, USA) were added to the mixture, which was incubated on ice for 30 minutes. The mixture was passed through a μ Column (Miltenyi Biotec) subjected to a magnetic field (μMACS separator; Miltenyi Biotec). The μ Column was rinsed four times with 200 μL of lysis buffer (CelLytic^™^ MT Cell Lysis Reagent; Sigma-Aldrich) and four times with 100 μL of Tris HCl pH 7.5. The bound protein was eluted from the column by adding 20 μL of elution buffer, and after 10 minutes adding another 50 μL of elution buffer and collecting the flow-through fraction. The elution buffer contained 0.2% sodium dodecyl sulfate and 0.1% Tween-20 in 50 mM Tris HCl, pH 8, to optimize recovery of immunoprecipitated proteins while minimizing elution of immunoglobulins bound to protein A [18].

The eluate was analyzed for SALSA by protein electrophoresis and Western blot. Fifteen μL of the eluate was combined with 10 μL of 4x Laemmli buffer supplemented with dithiothreitol and was separated by electrophoresis in a 12% SDS-polyacrylamide gel (TGX FastCast Acrylamide Solutions; Bio-Rad, Mississauga, ON, Canada). Separated proteins were visualized using a colorimetric stain (Imperial^™^ Protein Stain; Thermo Fisher Scientific, Mississauga, ON, Canada) per the manufacturer’s instructions. Duodenal tissue lysate was used as a positive control. A Western blot was performed to confirm the identity of the eluted protein, as previously described [12]. Immunoprecipitation was repeated as needed, and the protein was concentrated using Amicon^®^ Ultra-2 centrifugal filters (100 000 KDa; MilliporeSigma, Etobicoke, ON, Canada) per the manufacturer’s instructions. The concentration of the eluted protein was quantified using the Pierce^™^ Coomassie Plus protein assay (Thermo Fisher Scientific).

### Phagocytosis assay

Blood was collected into EDTA- or heparin-containing vacutainer tubes from 7 clinically healthy adult horses. These horses did not have any evidence of disease on physical examination, and had no abnormalities on routine prior bloodwork performed twice a year. Four horses had fecal examinations in the past year, with no abnormalities noted. Additional information regarding these horses is available in S1 Table. All animal procedures were approved by the University of Guelph Animal Care Committee (animal use protocol 4185), and conducted in accordance with guidelines from the Canadian Council on Animal Care. No general anesthesia, euthanasia, or any kind of animal sacrifice was part of the study. To investigate phagocytosis, bioparticles (pHrodo^™^ Green *Staphylococcus aureus* Bioparticles, Thermo Fisher Scientific) that fluoresce only after phagocytosis at the acidic pH of phagosomes were used. In duplicate flow cytometry tubes, 5 μL of bioparticles were incubated with either 5 μL of 20, 10, 5, or 2.5 μg/mL SALSA, or with 5 μL of PBS, for 30 minutes at 37°C in a water bath. Twenty-five μL of whole blood was then added to all tubes to allow phagocytosis, with one tube placed on ice and the other at 37°C in a water bath for 15 minutes. This represented a ratio of approximately 20 bacteria per neutrophil. Control tubes containing whole blood without bioparticles were treated similarly. After incubation, tubes in the water bath were placed on ice to stop phagocytosis. To each tube, 350 μL of 1x lysis buffer was added to lyse erythrocytes (10x lysis buffer contained 8.02 mg NH_4_Cl, 1 g KHCO_3_ and 100 μL of 0.5 M EDTA in 95 mL of ultrapure water). The samples were vortexed briefly, then incubated for 5 minutes at room temperature. One mL of flow buffer was added to each tube, and tubes were vortexed briefly. Flow buffer was prepared by combining 490 mL of PBS, 5 mL of 0.5 M EDTA, 5 mL of horse serum, and 0.065 g sodium azide, and adjusted to pH 7.4. The samples were centrifuged at 350 x g for 10 minutes, the supernatant was discarded, and for each tube the pellet was re-suspended in 1 mL of flow buffer. Centrifugation at 350 x g was repeated, the supernatant was discarded, and cell pellets were re-suspended in 0.5 mL of flow buffer. To assess cell viability, 5 μL of 7-aminoactinomycin D (7-AAD) was added to each tube.

Phagocytosis was analyzed using a flow cytometer (BD Accuri^™^ C6 Plus; BD, Mississauga, ON, Canada) and data analysis was performed using FlowJo 10.7.2 (BD). Leukocytes were identified by forward and side scatter cytograms, and fluorescence was collected for 10,000 events in the neutrophil gate. Fluorescent bioparticles were excited with a 488 nm laser and phagocytosis was measured using the 533/30 nm filter on a log scale. Cellular viability was assessed using the 670 LP filter. The degree of phagocytosis was assessed by measuring the percentage of viable neutrophils that emitted fluorescence at 533 nm relative to neutrophils that were not incubated with fluorescent bacteria for gating.

### Cytokine assay

Five clinically healthy female Standardbred horses, with no history of lung disease, were selected from a research herd. Information regarding the sex, breed, and age of these horses is provided in S2 Table. Horses were kept on pasture with indoor access, fed grass hay, given free access to water, and transported 7 km on the day of sample collection. Physical examination and endoscopy were performed, and no abnormalities were detected. A complete blood count and serum biochemistry were performed at the start of the study, and the findings were largely unremarkable: horses 2, 4, and 5 had a mild, less than 2-fold increase in glutamate dehydrogenase (range for all horses 5–12 U/L; reference interval 1–7 U/L). Serum amyloid A concentrations were zero in all horses. Bronchoalveolar lavage (BAL) was performed under standing sedation with 0.4–0.5 mg/kg xylazine (Bimeda, Cambridge, ON, Canada) and 0.01–0.03 mg/kg butorphanol (Zoetis, Kirkland, PQ, Canada) administered intravenously. Approximately 500 mL of sterile PBS was infused in order to retrieve at least 200 mL of BAL fluid (BALF) from each horse, as previously described [13]. The BALF samples were immediately placed on ice and processed within 20 minutes of collection. Differential BALF cell counts for each horse are provided in S3 Table.

**Fig 1:**
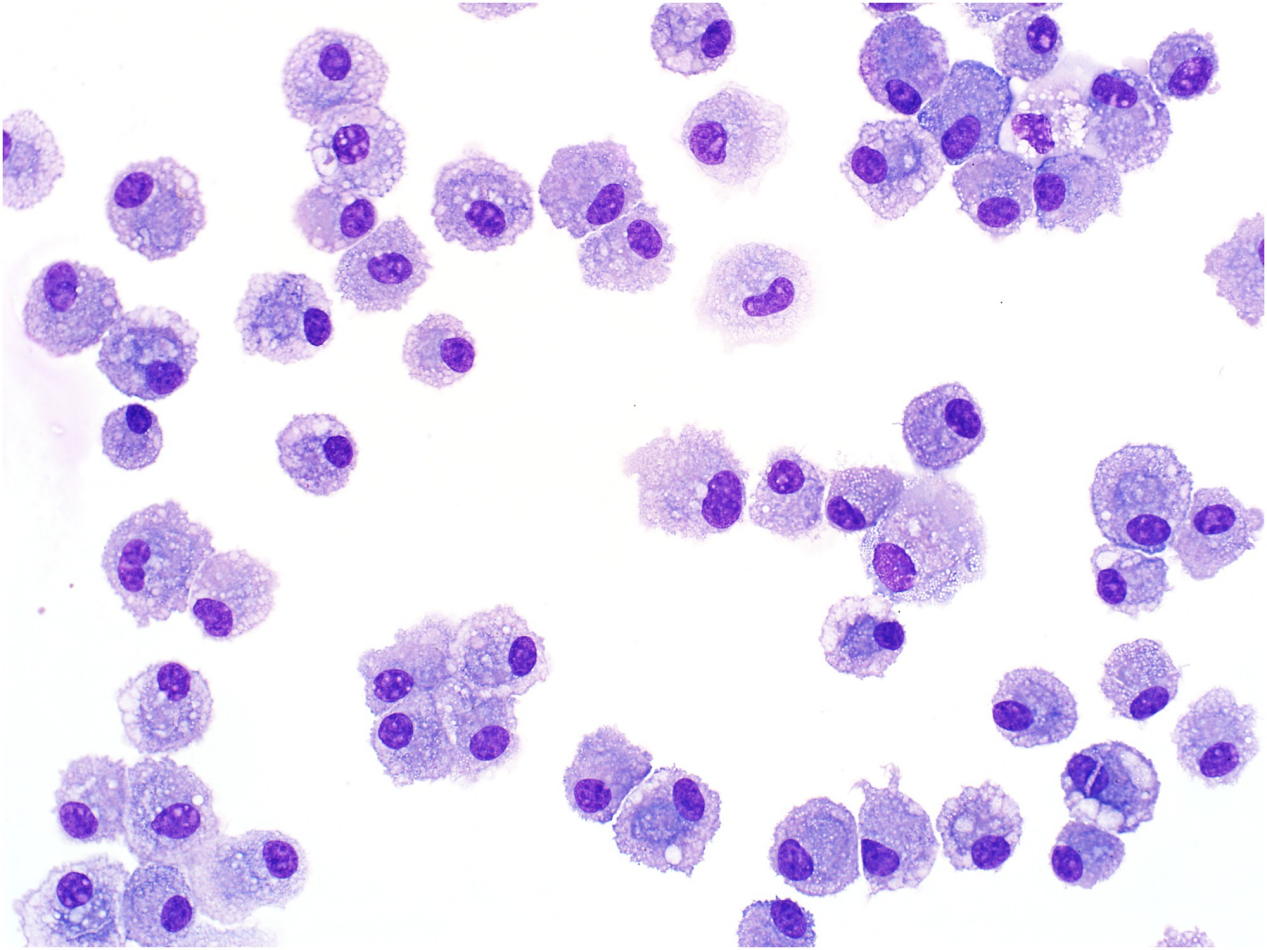
Cytocentrifuge preparation of alveolar macrophages after 7 days of culture. Bronchoalveolar lavage fluid cells were allowed to adhere for 4 hours, non-adherent cells were removed, and the remaining cells were cultured for 7 days. At that time point the cultures consisted of a uniform population of macrophages. Modified Wright’s stain, 400X magnification.

The BALF was centrifuged at 400 x g for 10 minutes, the supernatant was removed, and the cell pellet was washed three times in sterile PBS. Cells were resuspended in complete cell culture medium (RPMI 1640; Gibco^™^, Thermo Fisher Scientific), including penicillin-streptomycin (Gibco^™^, Thermo Fisher Scientific), β-mercaptoethanol (Bio-Rad) and 10% horse serum (Gibco^™^, Thermo Fisher Scientific). Approximately 10^8^ cells were seeded into a 75 cm^2^ cell culture flask (Nunc^™^, Thermo Fisher Scientific) and cultured at 37°C, 5% CO_2_ for 4 hours. The supernatant containing non-adherent cells was then removed and replaced with fresh complete cell culture medium. The media was replaced every two days for up to 7 days. Adherent cells were detached (TrypLE^™^ Select Enzyme (10X), Thermo Fisher Scientific) from the flasks and counted (Moxi Z Mini Automated Cell Counter, ORFLO Technologies). Microscopic evaluation of detached cells revealed a uniform population of macrophages (Fig 1). For each horse, 10 wells of a 96-well plate were seeded with 2 x 10^5^ macrophages in 200 μL of complete cell culture medium, and the empty remaining wells were filled with a similar volume of PBS to limit evaporation in adjacent wells. After overnight incubation at 37°C and 5% CO_2_, cell culture supernatant was removed and replaced with 100 μL of complete cell culture medium without serum to prevent interference of serum proteins with the cytokine assays. In duplicates, macrophages from these wells were split into five different incubation groups: the culture medium plus 20 μg/mL purified SALSA + 1 μg/mL LPS (Invitrogen^™^, eBioscience^™^, Thermo Fisher Scientific), 10 μg/mL purified SALSA + 1 μg/mL LPS, 5 μg/mL purified SALSA + 1 μg/mL LPS, 1 μg/mL LPS, or only media. The plate was incubated at 37°C, in 5% CO_2_ for 24 hours. The supernatant was then harvested and centrifuged at 1000 x g for 10 minutes. Incubation of macrophages from the different incubation groups for 24 hours resulted in >90% cellular viability, when assessed by Trypan blue exclusion. Differences in cellular viability with increasing concentrations of SALSA were not identified.

Analysis of cytokines in cell supernatant was performed with a Bio-Plex^®^ 200 Multiplex Immunoassay system (Bio-Rad), using the equine-specific Milliplex^®^ MAP Magnetic Bead Panel (MilliporeSigma, Burlington, MA, USA), per the manufacturer’s instructions. The standards provided for each cytokine and chemokine, as well as control materials, were suspended in serum-free cell culture medium. All samples were assayed in triplicate, and a minimum of 50 beads per well were analyzed. A total of 13 cytokines involved in innate and adaptive immunity were assessed: C-C motif chemokine ligand 2 (CCL2, also known as MCP-1), C-X-C motif chemokine ligand 1 (CXCL1, also known as GRO), CXCL10 (also known as IP-10), granulocyte colony-stimulating factor (G-CSF), granulocyte-macrophage colony-stimulating factor (GM-CSF), IL-1α, IL-1β, IL-6, IL-8, IL-10, IL-12, IL-18, and tumor necrosis factor alpha (TNF-α).

Data analysis was performed using the Belysa^™^ analysis software (MilliporeSigma). Measured mean fluorescence intensity (MFI) was plotted on a five-parameter logistical regression of the standard curve to derive analyte concentrations. Values that fell below the lower limit of detection were assigned half of the lowest concentration detectable by the assay.

### Statistical analysis

Analyses were performed using SAS version 9.4 (Cary, NC, USA). For the phagocytosis assay, a two-factor factorial in a randomized complete block design (RCBD) was used, with individual horses as a random block and fixed-effect factors being incubation group and temperature of incubation. The data were binary so were logit-transformed.

For the cytokine assay analysis, an RCBD with subsampling, to account for triplicates, was used with incubation group being the fixed-effect and individual horses being the random block.

Residual analyses were performed for both sets of data to assess ANOVA assumptions. This included testing for normality with Shapiro-Wilk, Kolmogorov-Smirnov, Cramer-von Mises, and Anderson-Darling tests, and plotting the residuals against both predicted values and explanatory variables used in the model. With respect to the cytokine assay, such residuals may have revealed outliers, unequal variants or the need for data transformation. Statistical significance was set at p ≤ 0.05.

## Results

### Purification of SALSA

**Fig 2:**
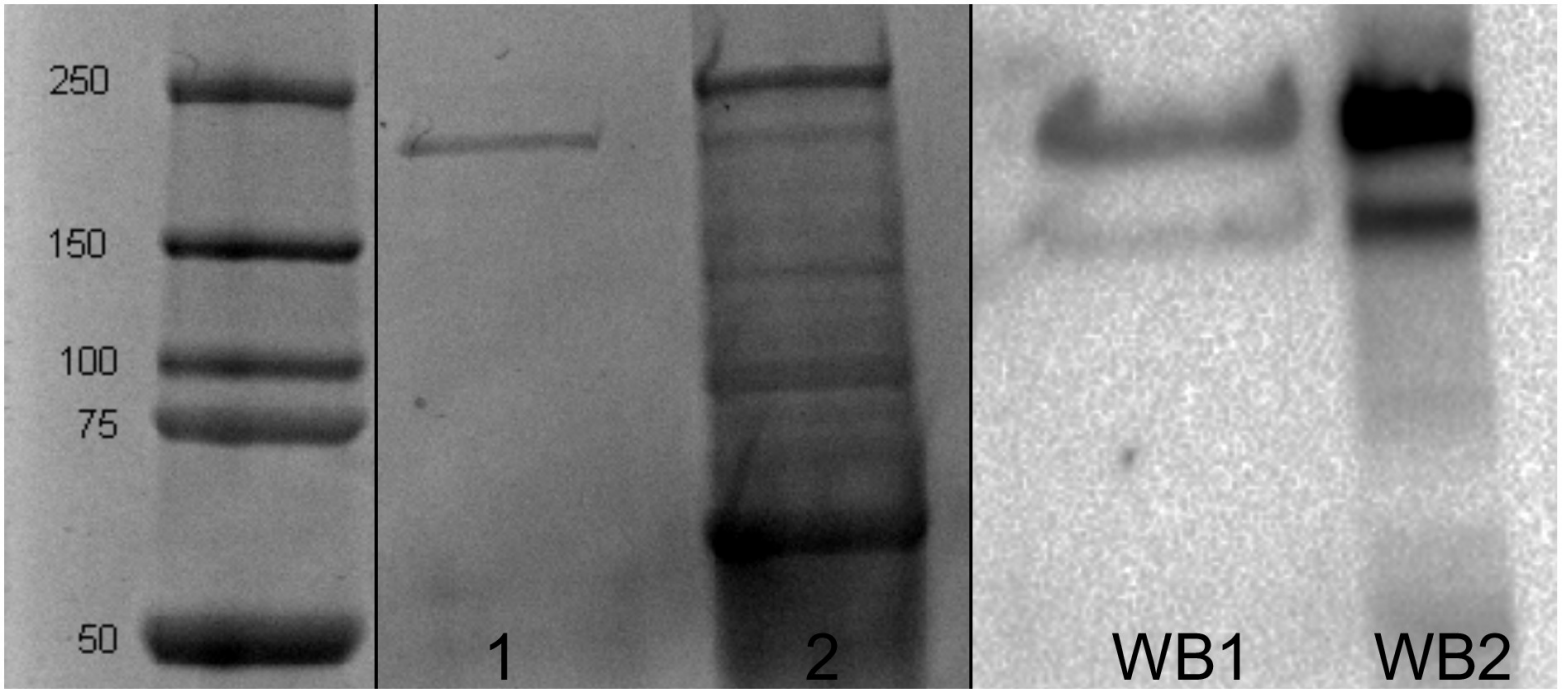
Electrophoresis and Western blot. Lanes 1 and 2: Protein electrophoresis. Lane 1: Eluate in buffer containing 0.2% sodium dodecyl sulfate and 0.1% Tween-20 in 50 mM Tris HCl pH 8 yielded a single band ~240 kDa, consistent with SALSA. Lane 2: Equine duodenal tissue lysate. Equine duodenal tissue lysate showed a prominent band of similar size. Lanes 1 and 2 were present on the same gel as the ladder, and these lanes were spliced together to only show the relevant lanes.

Protein electrophoresis of the eluate following SALSA immunoprecipitation revealed presence of an approximately 240 kD protein (Fig 2). The subsequent Western blot revealed a band of similar molecular weight, confirming specificity of protein immunoprecipitation. The size of the eluted protein matches prior reports of equine SALSA [12, 16].

Lanes WB1 and WB2: Western blot of eluate and duodenal tissue lysate, respectively. A protein of ~240 kD was detected with antibody to SALSA in both the eluate and the tissue lysate. A less intense lower molecular weight band likely represents an isoform of equine SALSA or degradation within these lanes.

### Phagocytosis assay

**Fig 3:**
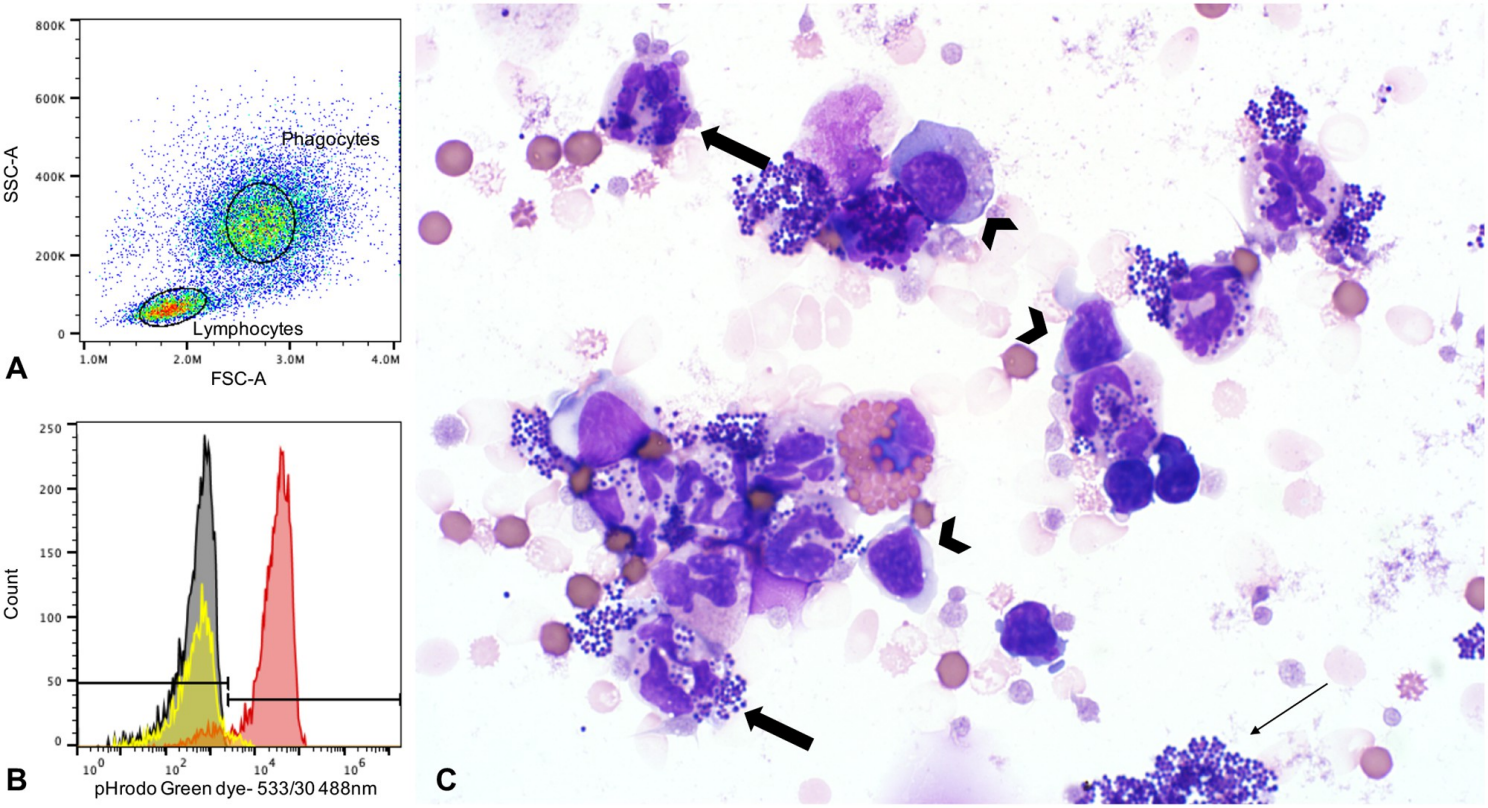
Phagocytosis assay. A: Phagocytes, which were mostly neutrophils, were gated based on high forward and side scatter. Lymphocytes were readily identified by low forward and side scatter. B: Fluorescence in three different cell populations; histograms. Neutrophils not incubated with bacteria (grey) were used to set the threshold for fluorescence. After incubation with bacteria, >90% of neutrophils (red) were fluorescent. Lymphocytes incubated with bacteria (yellow) did not fluoresce except for a small proportion (<4%), which may reflect inclusion of some phagocytes (monocytes or neutrophils) in the gate. C: Cytocentrifuge preparation of a sample incubated with 20 μg/mL SALSA at 37°C. Most neutrophils (thick arrows) contained multiple phagocytosed bacterial cocci in the cytoplasm. These bacteria were also noted in clusters extracellularly (thin arrow). Lymphocytes (arrowheads) did not contain bacteria. The background contains many lysed and a few intact erythrocytes, an expected finding in a sample that underwent erythrocyte lysis during sample preparation. Modified Wright’s stain, 1000X magnification.

The phagocytosis of bacteria was identified by an increase in fluorescence at 533 nm. Fluorescence was bimodal, with populations of cells that were either fluorescent, or non-fluorescent similar to cells not incubated with bacteria. Therefore, the percentage of fluorescent cells was used for analysis and assessment of phagocytosis (Table 1). Gating on neutrophils incubated with bacteria revealed an increase in fluorescence, indicating an increase in phagocytosis, which was confirmed by microscopic evaluation (Fig 3). On microscopic evaluation, differences in bacterial agglutination between the different incubation groups were not identified.

**Table 1:** Neutrophil phagocytosis of bacteria after incubation with different concentrations of SALSA.

Incubation group		Percent fluorescent neutrophils (37°C)						
	Horse	1	2	3	4	5	6	7
SALSA 20 μg/mL		63.4	83.6	49.8	85.1	93.5	69.4	97.7
SALSA 10 μg/mL		88.8	90.2	63.3	90.5	95.4	72.7	98.1
SALSA 5 μg/mL		85.2	91.0	60.6	91.4	91.3	67.9	97.9
SALSA 2.5 μg/mL		84.1	88.0	64.4	92.2	92.4	67.3	98.9
PBS		76.4	91.5	78.4	91.9	96.2	68.0	98.3
		Percent fluorescent neutrophils (ice)						
SALSA 20 μg/mL		61.8	84.6	65.8	81.9	95.5	72.6	97.0
SALSA 10 μg/mL		58.2	84.1	41.5	84.3	92.3	64.8	96.1
SALSA 5 μg/mL		70.0	89.8	57.8	85.1	95.2	74.0	95.5
SALSA 2.5 μg/mL		57.0	85.1	58.5	83.4	95.7	55.4	95.4
PBS		56.9	89.0	65.7	84.8	96.8	53.6	95.2

PBS: Phosphate buffered saline.

**Fig 4:**
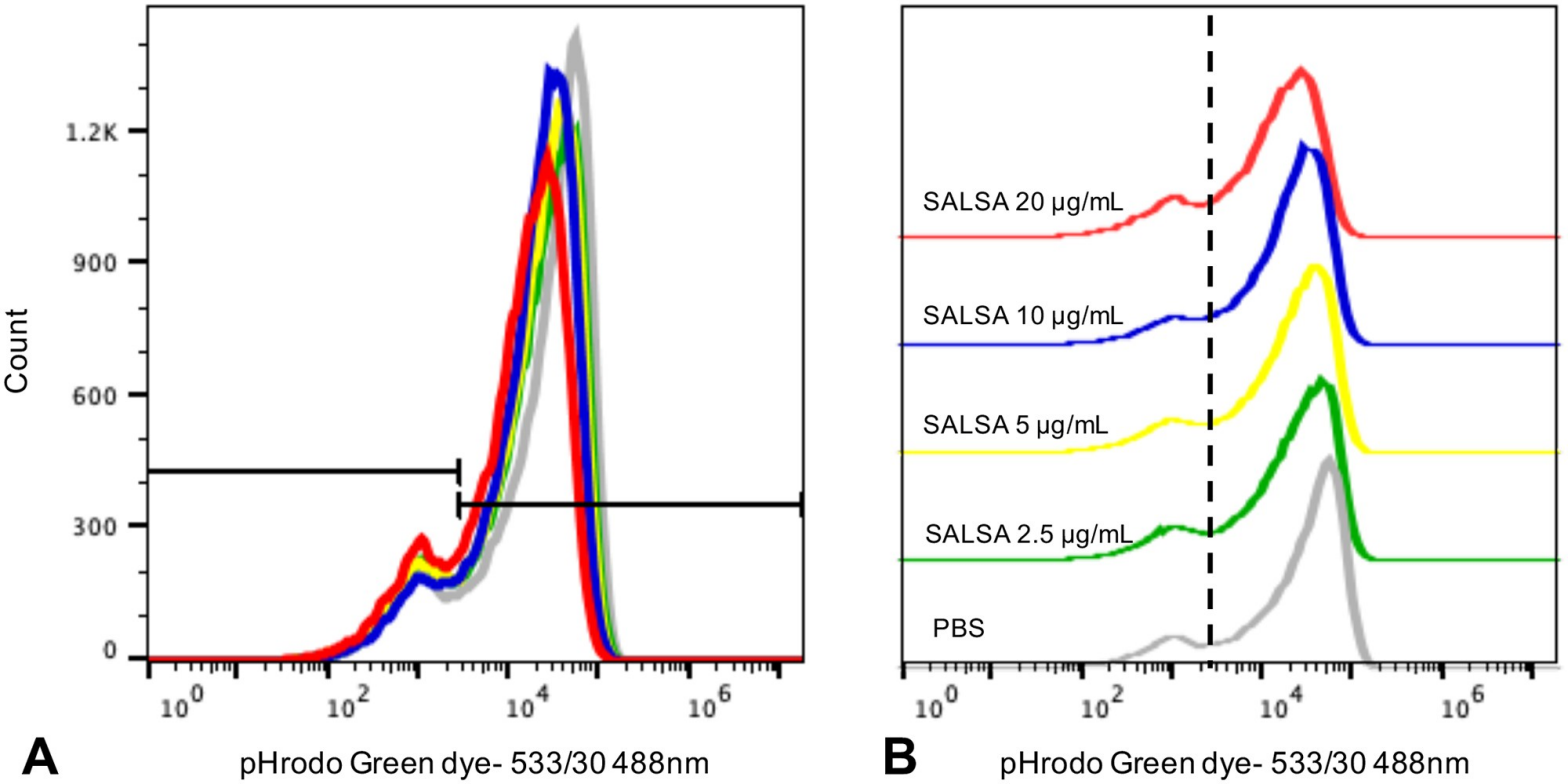
Neutrophil phagocytosis of bacteria after incubation with different concentrations of SALSA: A: The highest concentration of SALSA (red line, 20 μg/mL) resulted in fewer fluorescent and more non-fluorescent cells, consistent with decreased phagocytosis, compared to the other incubation groups. B: Ridgeline plot of fluorescence in cells incubated with different concentrations of SALSA or PBS.

The percentage of fluorescent neutrophils was significantly higher in samples incubated at 37°C compared to samples on ice (p < 0.0001). For the samples incubated on ice, there was no significant difference between samples incubated with different concentrations of SALSA or PBS in the percentage of fluorescent neutrophils. However, for the samples incubated at 37°C, incubation with 20 μg/mL SALSA yielded significantly lower percentages of fluorescent neutrophils compared to samples incubated with 10 μg/mL SALSA (p = 0.0078), 2.5 μg/mL SALSA (p = 0.0265), and PBS (p = 0.0048). The percentage of fluorescent neutrophils was also lower compared to samples incubated with 5 μg/mL SALSA, but the difference was not statistically significant (p = 0.0649) (Fig 4). There was no significant difference between samples incubated with 10, 5, and 2.5 μg/mL SALSA, and PBS (Table 2).

**Table 2:** P-values comparing fluorescent neutrophil percentages in samples incubated with different concentrations of SALSA.

Incubation group	SALSA 20	SALSA 10	SALSA 5	SALSA 2.5	PBS
SALSA 20	On ice	0.1234	0.8131	0.2887	0.6835
	37°C				
SALSA 10	**0.0078**	On ice	0.0768	0.6251	0.2539
		37°C			
SALSA 5	0.0649	0.3899	On ice	0.1958	0.5200
			37°C		
SALSA 2.5	**0.0265**	0.6378	0.6958	On ice	0.5115
				37°C	
PBS	**0.0048**	0.8626	0.3025	0.5201	On ice
					37°C

P-values below the diagonal line represent samples incubated at 37°C, and p-values above the diagonal line represent samples incubated on ice.

SALSA 20: 20 μg/mL SALSA

SALSA 10: 10 μg/mL SALSA

SALSA 5: 5 μg/mL SALSA

SALSA 2.5: 2.5 μg/mL SALSA

PBS: Phosphate buffered saline.

### Cytokine assay

Depending on the cytokine, some data were normal, some became normal after transformation, and others remained skewed despite transformation. Therefore, the statistical analysis differed by cytokine. For CCL2, CXCL10, G-CSF, IL-1β, IL-12, and TNF-α, the data were normal and did not require transformation. For CXCL1, IL-8, and IL-10, the data were log-transformed for analysis.

**Fig 5:**
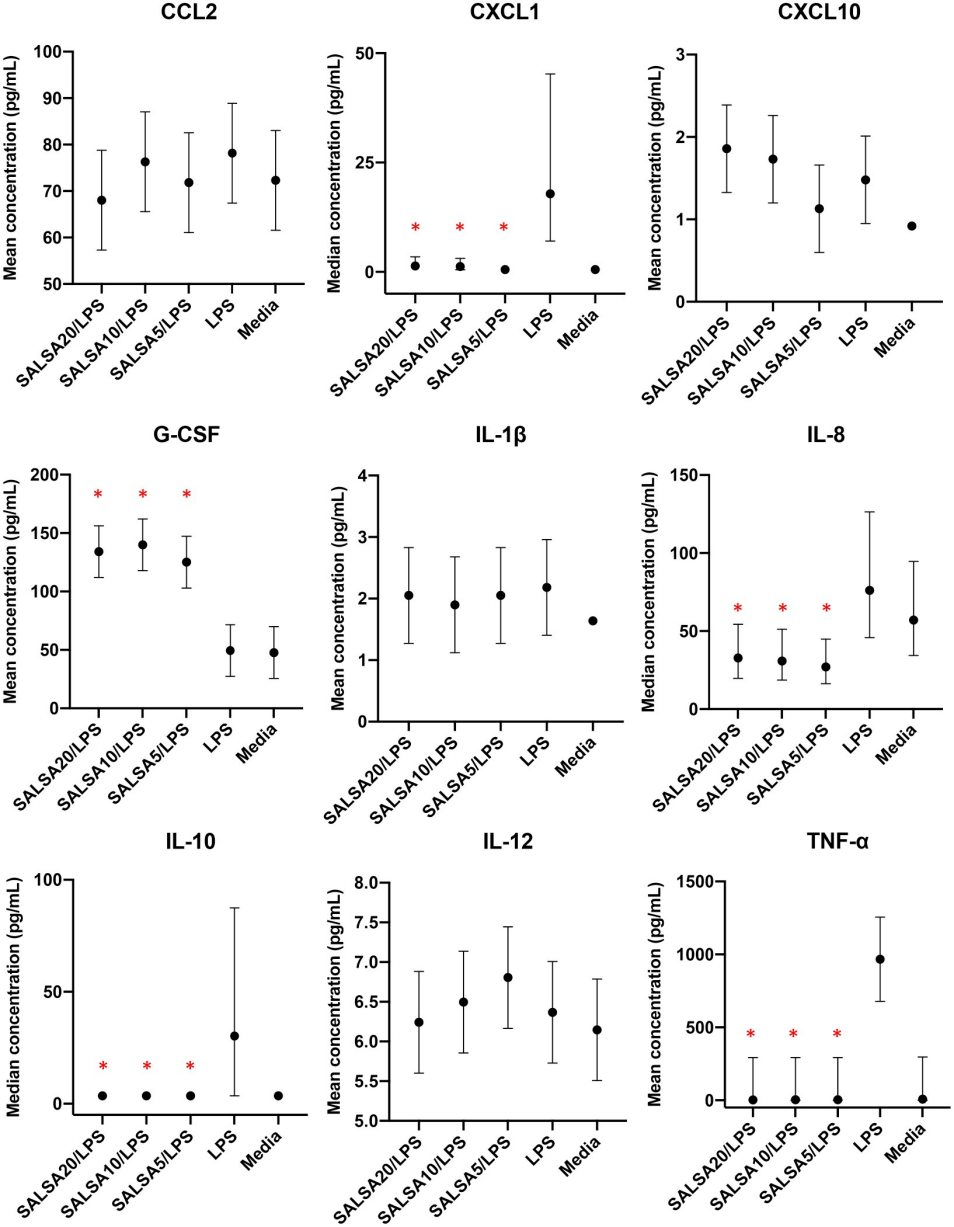
Mean or median cytokine concentration in alveolar macrophage supernatant. Macrophages were incubated for 24 hours with 20, 10, or 5 μg/mL of SALSA / 1 μg/mL LPS, with only LPS, or with media alone. Error bars represent the 95% confidence intervals. Red asterisks indicate samples incubated with SALSA which had a significant treatment effect compared to samples incubated with LPS alone.

Incubation with any concentration of SALSA was associated with a significant increase in G-CSF and a profound reduction in CXCL1, IL-8, IL-10, and TNF-α concentration relative to incubation with LPS (Table 3, Fig 5, S13 Table). As expected, IL-8 was high in supernatant of macrophages incubated with LPS. Incubation with SALSA resulted in a significant decrease in IL-8. Differences in CXCL10 and IL-1β concentration between macrophages incubated with 20, 10, or 5 μg/mL SALSA/LPS, and macrophages incubated with LPS alone, were not significant. Measurements of the four remaining cytokines, GM-CSF, IL-1β, IL-6, and IL-18, were all below the limit of detection (S1 File).

**Table 3:** Cytokine concentrations (pg/mL) in supernatant of alveolar macrophages.

Cytokine	Incubation	Mean	SD^a^	Median	25^th^ pct^b^	75^th^ pct
CCL2	SALSA 20/LPS^c^	68.04	17.19	80.30	49.65	80.30
	SALSA10/LPS	76.30	6.70	79.28	71.33	79.79
	SALSA5/LPS	76.61	3.37	77.75	73.87	78.77
	LPS^d^	78.15	1.32	77.75	77.23	79.28
	Media	72.30	13.31	78.26	63.37	78.26
CXCL1	SALSA 20/LPS^e^	1.20	0.61	1.14	0.65	1.78
	SALSA10/LPS^e^	0.94	0.51	0.78	0.52	1.44
	SALSA5/LPS^f^	0.75	0.52	0.52	0.52	1.11
	LPS	29.20	17.99	37.88	11.65	42.41
	Media	0.52	0.00	0.52	0.52	0.52
CXCL10	SALSA 20/LPS	6.92	2.60	6.11	5.13	9.12
	SALSA10/LPS	6.11	2.53	5.73	4.01	8.39
	SALSA5/LPS	3.29	2.13	2.39	2.15	4.88
	LPS	5.21	4.08	6.49	0.92	8.86
	Media	0.92	0.00	0.92	0.92	0.92
G-CSF	SALSA 20/LPS^e^	134.13	30.36	121.56	114.60	159.90
	SALSA10/LPS^e^	139.99	17.36	146.07	123.30	153.7
	SALSA5/LPS^e^	125.10	21.29	124.51	104.30	146.2
	LPS	49.51	19.16	42.67	35.76	66.68
	Media	47.77	26.09	42.67	26.75	71.33
IL-1β	SALSA 20/LPS	9.42	6.62	5.27	4.78	16.13
	SALSA10/LPS	7.64	6.42	8.17	1.95	13.06
	SALSA5/LPS	11.22	11.26	4.94	2.58	23.01
	LPS	11.43	11.08	8.72	4.47	19.75
	Media	1.97	0.73	1.64	1.64	2.46
IL-8	SALSA 20/LPS^e^	38.62	17.82	45.38	22.44	51.43
	SALSA10/LPS^e^	35.83	19.91	38.90	18.02	52.10
	SALSA5/LPS^e^	29.92	15.06	23.40	18.57	44.53
	LPS	77.87	16.14	80.93	65.42	88.77
	Media	59.71	16.85	65.38	46.58	70.01
IL-10	SALSA 20/LPS^f^	3.54	0.00	3.54	3.54	3.54
	SALSA10/LPS^f^	3.54	0.00	3.54	3.54	3.54
	SALSA5/LPS^f^	3.54	0.00	3.54	3.54	3.54
	LPS	32.70	34.44	30.27	3.54	63.07
	Media	3.54	0.00	3.54	3.54	3.54
IL-12	SALSA 20/LPS	6.24	0.44	6.12	5.88	6.67
	SALSA10/LPS	6.50	0.88	6.59	5.72	7.22
	SALSA5/LPS	6.81	0.32	6.91	6.50	7.07
	LPS	6.37	0.66	6.11	5.80	7.07
	Media	6.15	0.67	6.12	5.64	6.67
TNF-α	SALSA 20/LPS^e^	2.38	1.56	1.27	1.27	4.05
	SALSA10/LPS^e^	2.82	2.06	2.23	1.27	4.67
	SALSA5/LPS^e^	2.92	3.70	1.27	1.27	5.40
	LPS	967.15	682.70	1102.69	334.90	1532.00
	Media	7.09	6.21	4.62	1.75	13.65

^a^ Standard deviation;

^b^ Percentile;

^c^ SALSA 20 μg/mL + LPS 1 μg/mL;

^d^ LPS 1 μg/mL;

^e^ Samples incubated with SALSA that had statistically significant differences from samples incubated with LPS alone;

^f^ Samples incubated with SALSA for which data were not analyzed since at least one of the samples had cytokine concentrations below the limit of detection, but were considered significantly different from samples incubated with LPS alone.

## Discussion

Findings in this study show that neutrophils incubated with SALSA had decreased bacterial phagocytosis, and that macrophages incubated with SALSA had markedly altered cytokine production. These are novel effects of SALSA on leukocytes that support prior findings regarding the roles of SALSA in modulating inflammation [1, 8].

Our results showed more phagocytosis in samples incubated with SALSA at 37°C compared to samples incubated on ice, which confirmed prior observations about the importance of temperature for phagocytosis [19]. In many samples, incubation on ice did not completely abrogate phagocytosis, which is likely due to the protocol requiring frequent additional incubation steps at room temperature, which could create inconsistent cool temperature. There was inter-individual variation in the degree of phagocytosis, which could reflect inherent differences in the leukocytic response of individual horses, or variations brought upon by the methodology. Albeit, the analyses included at least 10,000 neutrophils per sample, yielding robust statistical results (p < 0.0001).

It was hypothesized that SALSA may increase phagocytosis, presumably through agglutination of bacteria. A prior study showed that agglutination of bacteria by saliva, which is rich in SALSA, enhanced their removal by phagocytes, while dispersed bacteria had increased survival [9]. Individualization of bacteria allows complement evasion and improves bacterial survival [20]. However, in the current study, a decrease in phagocytosis was noted in neutrophils incubated with SALSA. The postulated effects of SALSA involve multiple mechanisms including alterations in bacterial conformation, interactions with binding partners such as immune defense proteins, as well as other uncharacterized effects [10]. Whether reduced phagocytosis results from altering the conformation of bacteria or an interaction with neutrophils was not discerned, but an effect on bacteria seems likely given that SALSA’s SRCR domains recognize and bind a wide range of microbial surface structures [2]. Different from a previous study that used the chain-forming *Streptococcus gordonii*, the current study used *S*. *aureus*, which naturally form aggregates [9]. Such physical properties may affect the size of bacterial units to be phagocytosed. It has been shown that the optimal particle size for phagocytosis is 2 to 3 μm (equivalent to 2 to 4 coccoid bacteria), and that larger clusters may be more difficult for neutrophils to phagocytose [21]. Most neutrophils had phagocytosed multiple *S*. *aureus*, and further aggregation may have negatively impacted on their ability to phagocytose bacteria. Although clear differences in bacterial agglutination were not identified between different incubation groups on microscopic evaluation, it is possible that visual estimation may not be sensitive enough to detect subtle changes in agglutination. The propensity for *S*.*aureus* to form aggregates also complicated assessment. Future studies might directly evaluate the relationship between agglutination and phagocytosis, using bacteria less prone to aggregate. Additionally, saliva is a complex mix of proteins including multiple antimicrobial factors that can interact with SALSA and may influence bacterial aggregation or enhance deposition of complement factors or IgA. Therefore, purified SALSA may not reflect the collective effect of all salivary constituents on neutrophil phagocytosis [9, 22].

For the cytokine assays, alveolar macrophages were chosen due to their pivotal role in regulating inflammation in the lower airways, because SALSA is an abundant protein in bronchial secretions that is likely metabolized by alveolar macrophages, and since SEA predominantly affects the lower airways. Incubating alveolar macrophages with LPS simulates the effect of bacterial infection on the innate immune system and mimics SEA, which is exacerbated by inhalation of LPS combined with fungal spores and particulate [17]. The multiplex equine assay employed here was useful because it allowed concurrent and sensitive quantification of multiple cytokines in a small volume [23–25]. LPS is recognized by TLR4 on macrophages, which initiates the recruitment of adaptor molecules such as MyD88 and TRIF, and drives distinct signaling pathways that each lead to independent but complementary activation of NF-κB, resulting in induction of multiple cytokines [26]. Many of these cytokines, such as CXCL1, CXCL10, IL-6, IL-8, IL-10, and TNF-α, are released by macrophages [27–29]. Stimulation of alveolar macrophages with LPS triggered a marked increase in CXCL1, CXCL10, IL-1β, IL-10, and TNF-α relative to macrophages exposed to media alone. Measured values in supernatant from cultures exposed to media alone for CXCL1, CXCL10, IL-1β, IL-10, and TNF-α, were all below the limit of detection and did not show any variance, which precluded statistical analyses, but marked differences were nevertheless readily visualized (Fig 5). There was a measurable concentration of IL-8 in unstimulated macrophages that increased after incubation with LPS. This is similar to findings in humans where alveolar macrophages constitutively secrete high levels of IL-8, while other neutrophil chemoattractants, such as CXCL1, are not constitutively produced by alveolar macrophages [27, 30].

Alveolar macrophages that were incubated with SALSA plus LPS had lower concentrations of CXCL1, IL-8, IL-10, and TNF-α compared to those incubated with only LPS. The mechanism by which SALSA inhibits the release of these cytokines remains to be elucidated but considering that both pro-inflammatory CXCL1, IL-8, and TNF-α, and anti-inflammatory IL-10 were reduced, SALSA may interfere with recognition of LPS by TLR4 on macrophages, or inhibit the NF-κB pathway, as suspected previously [8]. Release of these cytokines involves interlinked downstream signaling molecules such as MyD88, activator protein-1 (AP-1), and signal transducer and activator of transcription (STAT) 3, as well as interplay among TLR4, RIG-I, and NOD-like receptor proteins, such that interference early in signaling would inhibit release of all these cytokines [31–36]. Therefore, it can be deduced that airway secretions containing SALSA modulate the release of cytokines by alveolar macrophages, and play a role in maintaining an anti-inflammatory environment within the lungs. In humans, mucosal homeostasis shifted toward tissue repair and regeneration was attributed to SALSA [3, 8, 37]. Expression of SALSA is typically increased in inflammation, for example through IL-22, which leads to STAT3 tyrosine phosphorylation and NF-κB activation, indicating a homeostatic anti-inflammatory role during inflammation [38]. However, in SEA where a decrease in SALSA gene expression was noted, the anti-inflammatory effect may be diminished, enabling exacerbated inflammation [12].

Higher G-CSF concentrations were identified in the supernatant of alveolar macrophages incubated with SALSA. This cytokine is typically viewed as a regulator of neutrophil production, but also has roles in immune modulation by influencing stem cell, T cell, and dendritic cell functions [39]. In mice and people, macrophage pre-treatment with G-CSF attenuated the release of pro-inflammatory cytokines such as TNF-α, IL-1β, IL-12, and IFN-γ after stimulation with LPS [40, 41]. Anti-inflammatory effects of G-CSF were associated with a shift towards Th2 lymphocytes and M2 macrophages, and through blockade of LPS-induced cJun N-terminal kinase activation [39, 42, 43]. Although the functions of G-CSF on cytokine production in horses are uncertain, a similar shift in phenotype among leukocytes may occur. Here, increased G-CSF production following incubation with SALSA may dampen the release of inflammatory cytokines by alveolar macrophages. There was no significant difference in G-CSF concentration between macrophages incubated with LPS alone and macrophages with media. This suggests that G-CSF release involves a pathway different from that of cytokines such as IL-8 and TNF-α, which could explain the increase in G-CSF after incubation with SALSA rather than an inhibitory effect. Therefore, it is possible that SALSA in airway secretions primes alveolar macrophages for release of G-CSF, creating an anti-inflammatory milieu within the alveolus and minimizing the response to triggers such as LPS, in order to maintain lung homeostasis. Upregulation of G-CSF has not previously been associated or investigated with SALSA; therefore, these results provide novel insight into the immunomodulatory mechanisms of SALSA.

Limitations to this study pertain to the number of individuals, in particular for the cytokine assay. Nevertheless, for most cytokines, inter-individual differences were limited. Additional insight on the dynamics of cytokine production may have been gained from multiple measurements during the 24 hours of SALSA incubation since cytokine release may be transient. Different amounts of SALSA yielded apparent concentration-dependent changes for several cytokines (CCL2, CXCL10, IL-8, and IL-12) that would ideally be confirmed over a wider range of SALSA concentrations. One horse had 4% mast cells on BALF cytology, which is considered supportive of mild/moderate equine asthma (MEA) (S3 Table). Mild/moderate equine asthma is very common, affecting over 60% of horses on pasture [44]. Unlike SEA, MEA is often subclinical at rest, and BALF cytology is generally required for a diagnosis. A prior study using a multiplex cytokine assay similar to that in our study identified significant differences in inflammatory cytokines in neutrophilic MEA compared to healthy horses, but not between mastocytic MEA compared to healthy horses [44]. Therefore, the influence of this particular horse on the overall findings of this study is deemed minimal. This is supported by relatively similar cytokine concentration and responses to SALSA across all horses in this study. As mentioned above, 3 horses also had a mild increase in serum glutamate dehydrogenase. The cause for this increase is unclear, but the low magnitude, in combination with the normal physical examination findings, normal serum amyloid A concentrations, and relatively similar cytokine changes across all horses in response to LPS and LPS with SALSA, makes this unlikely to affect the aims of this study.

In summary, decreased bacterial phagocytosis by neutrophils incubated with SALSA may be one factor in a complex interplay between pro- and anti-inflammatory activities at mucosal sites. Neutrophils are quickly recruited to alveoli following loss of mucosal integrity, and exposure to SALSA may limit their phagocytic activity at such sites. This effect is in concordance with attenuation of pro-inflammatory cytokine production by alveolar macrophages. Hence, in vivo investigation of SALSA as an anti-inflammatory compound may be rewarding.

## Supporting information

S1 Fig Phagocytosis assay; comparing results of blood collected in EDTA versus heparin. A: Neutrophils were gated based on their relatively high forward and side scatter properties. B: Flow cytometric analysis of blood incubated without bacteria was used to set the threshold for fluorescence. Thresholds were consistent for blood collected in EDTA or heparin. C—F: Percentage of fluorescent neutrophils in blood collected in EDTA and heparin in different horses. Neutrophils collected in heparin had slightly higher fluorescence compared to those collected in EDTA; however, the differences were mild and proportional among different horses. Incubation temperature was 37°C.
(TIF)

S1 File Raw data for the cytokine assay. (XLSX)

S1 Raw images Raw images for protein electrophoresis (A) and Western blot (B). Images were captured using a Chemidoc^+^ instrument and ImageLab software (both Bio-rad, Mississauga, ON, Canada). Images were spliced together to form Fig 2. Crosses denote lanes that were not depicted in Fig 2. Loading order: left to right.
(PDF)

S1 Table Phagocytosis assay, horse information. (XLSX)

S2 Table Cytokine assay, horse information. (XLSX)

S3 Table Cytokine assay, bronchoalveolar lavage fluid cell differential counts. (XLSX)

S4 Table Cytokine concentrations, CCL2 (pg/mL). (XLSX)

S5 Table Cytokine concentrations, CXCL1 (pg/mL). (XLSX)

S6 Table Cytokine concentrations, CXCL10 (pg/mL). (XLSX)

S7 Table Cytokine concentrations, G-CSF (pg/mL). (XLSX)

S8 Table Cytokine concentrations, IL-1β (pg/mL). (XLSX)

S9 Table Cytokine concentrations, IL-12 (pg/mL). (XLSX)

S10 Table Cytokine concentrations, IL-8 (pg/mL). (XLSX)

S11 Table Cytokine concentrations, IL-10 (pg/mL). (XLSX)

S12 Table Cytokine concentrations, TNF-α (pg/mL). (XLSX)

S13 Table P-values comparing cytokine production by macrophages incubated with different concentrations of SALSA. (DOCX)

S14 Table Neutrophil phagocytosis of bacteria after incubation with different concentrations of SALSA, mean and median fluorescence intensity. (XLSX)
