# Supplementary figures and images for: Effects of equine SALSA on neutrophil phagocytosis and macrophage cytokine production
Source: PLoS One. 2022 Mar 14;17(3):e0264911. doi: 10.1371/journal.pone.0264911 (PMC8920288; doi:10.1371/journal.pone.0264911)

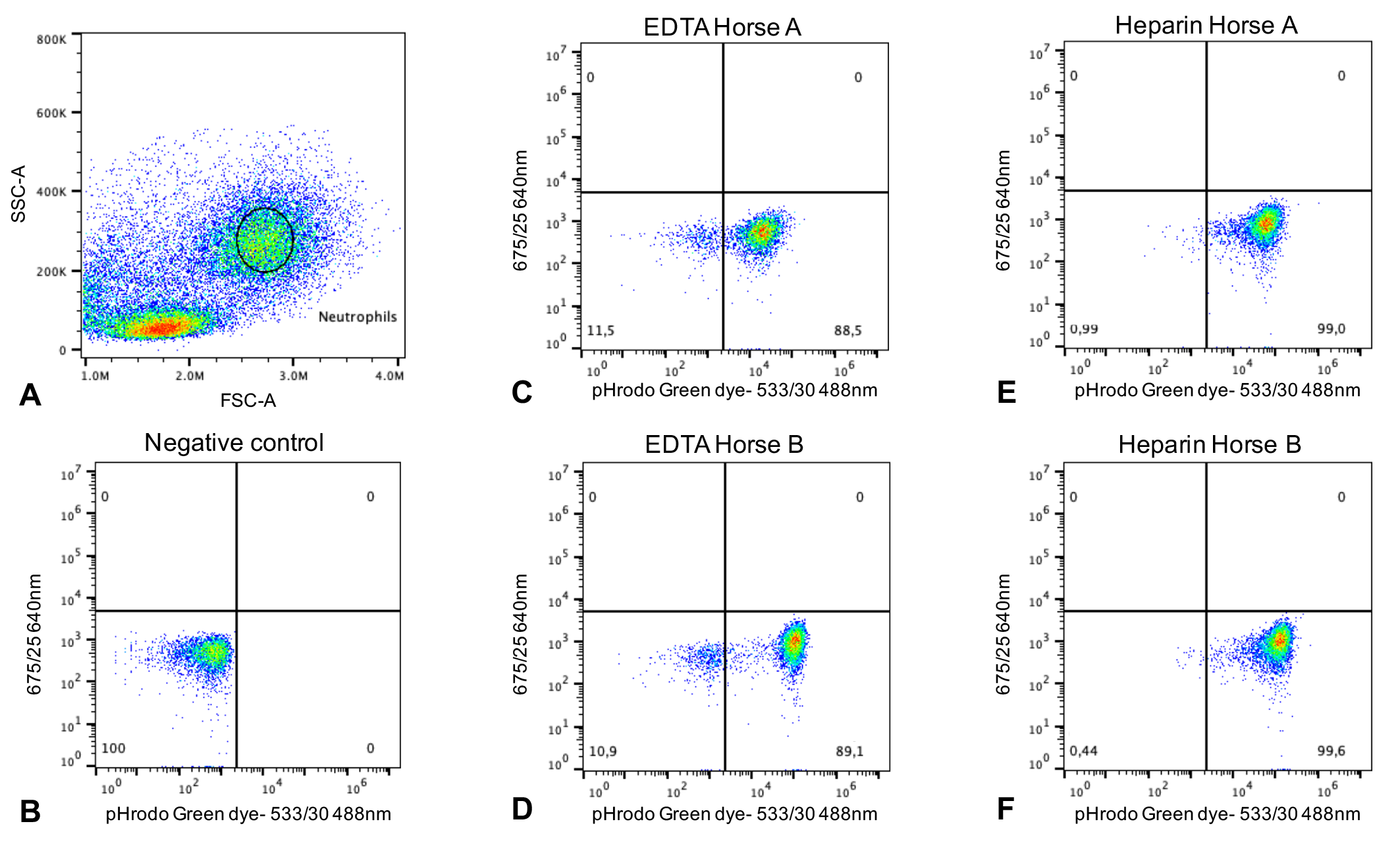

Supplement: S1 Fig — A: Neutrophils were gated based on their relatively high forward and side scatter properties. B: Flow cytometric analysis of blood incubated without bacteria was used to set the threshold for fluorescence. Thresholds were consistent for blood collected in EDTA or heparin. C—F: Percentage of fluorescent neutrophils in blood collected in EDTA and heparin in different horses. Neutrophils collected in heparin had slightly higher fluorescence compared to those collected in EDTA; however, the differences were mild and proportional among different horses. Incubation temperature was 37°C. (TIF) [file pone.0264911.s001.tif]

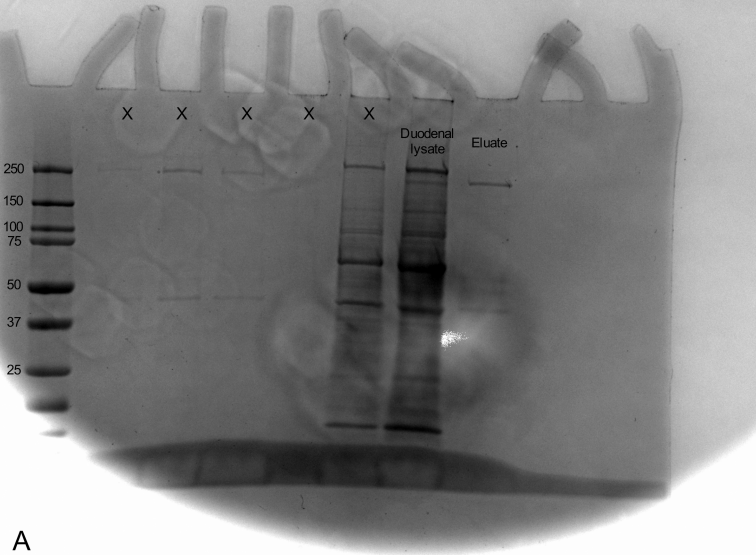

A

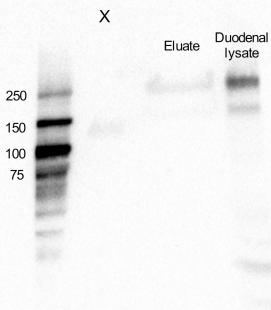

B

Supplement: S1 Raw images — Images were captured using a Chemidoc+ instrument and ImageLab software (both Bio-rad, Mississauga, ON, Canada). Images were spliced together to form Fig 2. Crosses denote lanes that were not depicted in Fig 2. Loading order: left to right. (PDF) [file pone.0264911.s003.pdf]
