# Supplementary material for: Effects of equine SALSA on neutrophil phagocytosis and macrophage cytokine production
Source: PLoS One. 2022 Mar 14;17(3):e0264911. doi: 10.1371/journal.pone.0264911 (PMC8920288; doi:10.1371/journal.pone.0264911)
Supplement: S13 Table — (DOCX) [file pone.0264911.s016.docx]

**Supplemental Table 13: P-values comparing cytokine production by macrophages incubated with different concentrations of SALSA**

| Cytokine | Incubation | SALSA20/LPS^a^ | SALSA10/LPS | SALSA5/LPS | LPS^b^ | Neg |
| --- | --- | --- | --- | --- | --- | --- |
| CCL2^d^ | SALSA20/LPS | - | 0.2087 | 0.5574 | 0.1283 | 0.5088 |
|  | SALSA10/LPS | 0.2087 | - | 0.4874 | 0.7728 | 0.5350 |
|  | SALSA5/LPS | 0.5574 | 0.4874 | - | 0.3301 | 0.9398 |
|  | LPS | 0.1283 | 0.7728 | 0.3301 | - | 0.3673 |
|  | Media | 0.5088 | 0.5350 | 0.9398 | 0.3673 | - |
| CXCL1^e^ | SALSA20/LPS | - | 0.8390 | n/a | **0.0012** | n/a |
|  | SALSA10/LPS | 0.8390 | - | n/a | **0.0010** | n/a |
|  | SALSA5/LPS | n/a^c^ | n/a | - | n/a | n/a |
|  | LPS | **0.0012** | **0.0010** | n/a | - | n/a |
|  | Media | n/a | n/a | n/a | n/a | - |
| CXCL10^d^ | SALSA20/LPS | - | 0.7195 | 0.0562 | 0.2944 | n/a |
|  | SALSA10/LPS | 0.7195 | - | 0.1065 | 0.4802 | n/a |
|  | SALSA5/LPS | 0.0562 | 0.1065 | - | 0.3294 | n/a |
|  | LPS | 0.2944 | 0.4802 | 0.3294 | - | n/a |
|  | Media | n/a | n/a | n/a | n/a | - |
| G-CSF^d^ | SALSA20/LPS | - | 0.6966 | 0.5496 | **<0.0001** | **<0.0001** |
|  | SALSA10/LPS | 0.6966 | - | 0.3283 | **<0.0001** | **<0.0001** |
|  | SALSA5/LPS | 0.5496 | 0.3283 | - | **0.0001** | **<0.0001** |
|  | LPS | **<0.0001** | **<0.0001** | **0.0001** | - | 0.9075 |
|  | Media | **<0.0001** | **<0.0001** | **<0.0001** | 0.9075 | - |
| IL-1β^d^ | SALSA20/LPS | - | 0.7317 | 0.9999 | 0.9901 | n/a |
|  | SALSA10/LPS | 0.7317 | - | 0.7317 | 0.5271 | n/a |
|  | SALSA5/LPS | 0.9999 | 0.7317 | - | 0.9901 | n/a |
|  | LPS | 0.9901 | 0.5271 | 0.9901 | - | n/a |
|  | Media | n/a | n/a | n/a | n/a | - |
| IL-8^e^ | SALSA20/LPS | - | 0.7986 | 0.4243 | **0.0022** | **0.0293** |
|  | SALSA10/LPS | 0.7986 | - | 0.5830 | **0.0013** | **0.0174** |
|  | SALSA5/LPS | 0.4243 | 0.5830 | - | **0.0004** | **0.0054** |
|  | LPS | **0.0022** | **0.0013** | **0.0004** | - | 0.2314 |
|  | Media | **0.0293** | **0.0174** | **0.0054** | 0.2314 | - |
| IL-10^e^ | SALSA20/LPS | - | n/a | n/a | n/a | n/a |
|  | SALSA10/LPS | n/a | - | n/a | n/a | n/a |
|  | SALSA5/LPS | n/a | n/a | - | n/a | n/a |
|  | LPS | n/a | n/a | n/a | - | n/a |
|  | Media | n/a | n/a | n/a | n/a | - |
| IL-12^d^ | SALSA20/LPS | - | 0.5597 | 0.2065 | 0.7728 | 0.8261 |
|  | SALSA10/LPS | 0.5597 | - | 0.4796 | 0.7664 | 0.4248 |
|  | SALSA5/LPS | 0.2065 | 0.4796 | - | 0.3202 | 0.1424 |
|  | LPS | 0.7728 | 0.7664 | 0.3202 | - | 0.6123 |
|  | Media | 0.8261 | 0.4248 | 0.1424 | 0.6123 | - |
| TNF-α^d^ | SALSA20/LPS | - | 0.9986 | 0.9968 | **0.0001** | 0.9825 |
|  | SALSA10/LPS | 0.9986 | - | 0.9982 | **0.0001** | 0.9840 |
|  | SALSA5/LPS | 0.9968 | 0.9982 | - | **0.0001** | 0.9858 |
|  | LPS | **0.0001** | **0.0001** | **0.0001** | **-** | **0.0001** |
|  | Media | 0.9825 | 0.9840 | 0.9858 | **0.0001** | - |

^a^ SALSA 20 μg/mL + LPS 1 μg/mL

^b^ LPS 1 μg/mL

^c^ Not applicable. Data were not analyzed since at least one of the compared groups had cytokine concentrations below the limit of detection.

^d^ Data were normally distributed, thus mean concentrations were compared.

^e^ Data were not normally distributed, thus median concentrations were compared.
